# EffHunter: A Tool for Prediction of Effector Protein Candidates in Fungal Proteomic Databases

**DOI:** 10.3390/biom10050712

**Published:** 2020-05-04

**Authors:** Karla Gisel Carreón-Anguiano, Ignacio Islas-Flores, Julio Vega-Arreguín, Luis Sáenz-Carbonell, Blondy Canto-Canché

**Affiliations:** 1 Unidad de Biotecnología, Centro de Investigación Científica de Yucatán, A.C., Calle 43 No. 130 X 32 y 34, Col. Chuburná de Hidalgo, Mérida C.P. 97205, Mexico; karla.carreon@cicy.mx (K.G.C.-A.); vyca@cicy.mx (L.S.-C.); 2 Unidad de Bioquímica y Biología Molecular de Plantas, Centro de Investigación Científica de Yucatán, A.C., Calle 43 No. 130 X 32 y 34, Col. Chuburná de Hidalgo, Mérida C.P. 97205, Mexico; islasign@cicy.mx; 3 Laboratorio de Ciencias AgroGenómicas, Escuela Nacional de Estudios Superiores-UNAM, León 37689, Mexico; jvega@enes.unam.mx

**Keywords:** computational prediction, host-pathogen interaction, effector proteins, fungal secretome

## Abstract

Pathogens are able to deliver small-secreted, cysteine-rich proteins into plant cells to enable infection. The computational prediction of effector proteins remains one of the most challenging areas in the study of plant fungi interactions. At present, there are several bioinformatic programs that can help in the identification of these proteins; however, in most cases, these programs are managed independently. Here, we present EffHunter, an easy and fast bioinformatics tool for the identification of effectors. This predictor was used to identify putative effectors in 88 proteomes using characteristics such as size, cysteine residue content, secretion signal and transmembrane domains.

## 1. Introduction

Fungal phytopathogens are a major threat to food security since they can cause devastating losses to important crops in agriculture.

These pathogenic fungi secrete diverse small proteins in the infection process which are pathogenicity or virulence determinants that manipulate the interaction, and thus are commonly referred to as “effectors” [1,2,3,4]. Small, secreted, cysteine-rich proteins constitute a common source of fungal effectors [3,5,6,7].

Fungal effector proteins are poorly conserved, and in contrast to oomycetes effectors, where the presence of conserved amino acid motifs (e.g., RxLR, dEER) has created profile Hidden Markov Models (HMM), the prediction of fungal effectors has been more challenging. In general, most fungal effectors do not share significant sequence similarity with each other, which can be attributed to rapid divergence and host specialization. However, they share structural properties such as a signal peptide for secretion, absence of transmembrane domains, presence of some motifs, small-medium molecular weight sizes and cysteine-rich content [8,9,10]. Additional fungal effector features have been reported for specific subclasses of effectors, for example, particular genomic locations such as gene clusters, gene-sparse regions or localization in dispensable chromosomes [11].

Major efforts have been devoted to in silico identification of secreted effectors in large-scale genome studies. Three principal approaches have been commonly used by different research groups: (a) analyzing proteomes in different bioinformatic programs that help to filter the secretome, for instance, SignalP 4.1 [12], WoLFPSORT [13] and TMHMM 2.0 [14]. The use of these programs is usually done in a separate manner and not as a package; (b) machine learning approaches, which can predict new effector proteins based on the extracted features of reported and confirmed effectors [15,16]; and (c) comparative genomics to search those effectors that belong to families, or to find rare ortholog effector candidates that might be transferred horizontally [17].

EffectorP 1.0 and 2.0, the first reported machine learning classifiers of fungal effectors [15,16], have been the most preferred fungal effector prediction tools used to date. Although these approaches are often successful at identifying effectors, criteria such as small size and enrichment in cysteine require thresholds manually set by researchers. Therefore, finding an easy and quick tool that led to adjusting the search criteria used for the prediction of effectors becomes a niche of opportunity.

The present work introduces EffHunter, a pipeline developed to integrate SignalP 4.1 [12], Phobius [18], TMHMM 2.0 [14] and WoLFPSORT [13] with Perl/Bioperl scripts for filtering protein size and cysteine content. Running the analysis in a single step, the sensitivity of the prediction was 70%, defined as the proportion of positives that are correctly identified, and a specificity of 100%, which is the proportion of negatives that are correctly identified. These values obtained were the same as those obtained with the step-by-step method of prediction and similar to EffectorP 2.0 values with a sensitivity of 68% and specificity of 98%.

## 2. Materials and Methods

### 2.1. Architecture of EffHunter Pipeline

EffHunter is based on the available software SignalP 4.1 [12], Phobius [18], TMHMM 2.0 [14] and WoLFPSORT [13] (Table 1). A set of Perl/Bioperl (v5.18.2) scripts was designed to perform these complementary tasks: the protein length analysis (≤400 amino acids), the cysteine count (≥4 cysteine residues) and connecting the individual steps into a single pipeline for proteome scale analysis. EffHunter works as follows: first, all the sequences are put in FASTA format, where each sequence is filtered with respect to the size indicated by the user, and the result is stored in a new FASTA file. Second, the first list obtained from the filter by protein size is submitted to the filter by number of cysteines; in this step, the user indicates the value to filter those proteins that have greater or equal to the number indicated. Once obtained, the proteins that meet the criteria are stored in a new FASTA file. Third, the retrieved sequences are searched for signal peptide signatures with SignalP 4.1 and Phobius programs. Fourth, the resulting FASTA file of protein sequences are searched for transmembrane domains with TMHMM 2.0, and then WoLFPSORT predicts the subcellular localization. Each analysis uses a FASTA file as input and generates an output in FASTA format too.

EffHunter is distributed as a compressed file in ZIP format. The source code is available for download at https://www.cicy.mx/unidad-de-biotecnologia/investigador/blondy-beatriz-canto-canche and https://github.com/GisCarreon/EffHunter_v.1.0 GitHub repository. Docker image is available at https://hub.docker.com/r/giscarreon/ubuntu-effhunter. Once the EffHunter_1.0 directory has been uncompressed, it shows the bin directory, in which the empty subdirectories SignalP, Phobius, TMHMM and WoLFPSORT are located. The user must download each program from the platforms indicated in Table 1 and uncompress and install them in each empty subdirectory mentioned above for the correct execution of EffHunter. To use EffHunter pipeline, the code indicates the necessary path to install each program and to compile and take the modules. The command to execute EffHunter once it is installed in a linux/unix terminal is *sh ./EffHunter.sh*.

### 2.2. Validation of EffHunter Pipeline in ab Initio Approach

EffHunter was challenged using different protein databases designated as positive and negative control sets. The positive data set (Table 2) contained a total of 150 effector proteins (Supplementary data set S1), of which 94 effectors were collected from the literature by Sperchneider et al. [15], and the other 56 were effector candidates retrieved from the Pathogen-Host Interaction database (PHI-base) (Supplementary Table S1). These databases comprise, to date, more than 4000 proteins involved in pathogenicity, from more than 260 plant and animal pathogens; 70% of them being phytopathogens [19,20]. The search for protein effectors in the PHI base for the positive data set was done using the following criteria: length ≤400 amino acids [5,21,22,23,24,25], ≥4 cysteine residues [26,27], presence of signal peptide and lack of transmembrane domains [1,27,28].

The capability of EffHunter pipeline to exclude non-effectors was challenged with a large list of negative control proteins (4530 proteins). The negative control set comprises well-known families of proteins, ABC transporter proteins (2329) (Supplementary data set S2) [29,30], Cyt P450 proteins (476) (Supplementary data set S3) [31], and 1725 proteins classified as major facilitator transporters (MFTS) (Supplementary data set S4) [32,33,34]. The set of negative controls comprises varied proteins: from 73 to 5000 amino acids; from 0 to 74 cysteines; from 0 to 23 TMDs; with or without signal peptide. The great variability makes these proteins a robust set of negative controls. In addition, none are extracellular, and more importantly, no member of these protein families has been described as a fungal effector. The fasta sequences of all of these candidates were downloaded from the GenBank at NCBI (https://www.ncbi.nlm.nih.gov/); positive and negative controls were pooled in a single database containing in total 4680 proteins (Supplementary data set S5).

The search for effectors in this database was performed in the traditional way using each program separately by sequential analyses and in a single step, using the EffHunter pipeline with the command described above.

To assess the predictive ability of the EffHunter prediction, the variables sensitivity, specificity, precision and accuracy were calculated to measure the performance of the EffHunter pipeline:

(1) $$Sensitivity/Recall\frac{TP}{\left(TP+FN\right)}$$

(2) $$Specificity=\frac{TN}{\left(TN+FP\right)}$$

(3) $$PPV/Precision=\frac{TP}{\left(TP+FP\right)}$$

(4) $$ACC=\frac{TP+TN}{\left(TP+FP+FN+TN\right)}$$

(5) $$FPR=\frac{FP}{\left(FP+TN\right)}$$

(6) $$F1=2\times \frac{precision\times recall}{precision+recall}$$

Sensitivity is defined as the proportion of positives that are correctly identified. Specificity is the proportion of negatives that were correctly identified. Precision or positive predictive value, PPV, is a measure which captures the proportion of positive predictions that are true. Accuracy analysis can be used to evaluate the overall performance of a method. In the equations, TP, true positives; TN, true negatives; FP, false positives; and FN, false negatives. Recall is defined as the proportion of the positives that are successfully retrieved. F1 Score is widely used to measure the success of binary classifier and compare performance of different software/pipelines. F1 Score reaches it best value at 1 and the worst score at 0 [16,25,28,35,36,37].

### 2.3. Validation of EffHunter Pipeline in Comparative Approach

Criteria to identify effectors are largely discrepant in the literature; therefore, in order to continue the evaluation of EffHunter, its predictions were compared with three published fungal effector datasets. Each dataset was obtained by using different strategies to identify the effectoromes.

Two approaches were followed. In the first one, we compared EffHunter prediction with previous reports of effector prediction, by sequential/separate analyses with different bioinformatics programs (Table 3). Reports were for *Blumeria graminis* f. sp. *hordei* [38], *Pseudocercospora fijiensis* [39] and *Mycosphaerella graminicola* [40]. It is important to emphasize that in those reports, different combinations of bioinformatic tools were used. Details about such tools are provided in Table 4.

For *Blumeria graminis* f. sp. *hordei* and *Pseudocercospora fijiensis*, EffHunter analyses ran on the same bioinformatic databases used by the authors, but for *Mycosphaerella graminicola*, EffHunter ran on the nonredundant dataset of proteins for this pathogen at JGI (https://mycocosm.jgi.doe.gov/Mycfi2/Mycfi2.home.html) because the database reported by authors is not publicly available. In the search for effectors, the length cutoff was set equal to that used in each one of these reports, respectively.

The second approach was a comparison between the resulting list of protein effectors produced by EffHunter with those of Sonah et al. [10], since the authors used the bioinformatics tool, SECRETOOL [41], a program that integrates the use of SignalP 4.1, TMHMM 2.0 and WoLFPSORT to analyze and identify secreted proteins. Using SECRETOOL, the authors predicted effectoromes in 12 proteomes: *Alternaria brassisicola, Blumeria graminis, Cladosporium fulvum, Colletrotrichum gramnicola, Fusarium oxysporum, Leptosphaeria maculans, Magnaporthe oryzae, Mycosphaerella graminicola, Ustilago maydis, Puccinia graminis* f. sp. *tritici, Pyrenophora tritici-repentis* and *Phytophthora infestans*. Comparison among in silico identification of effectors by EffHunter, SECRETOOL and EffectorP 2.0 was carried out for these pathogens. For this and further analyses, cutoff size was fixed at ≤400 amino acids and the cysteine residues ≥4 in EffHunter analyses.

As a final test for validation, the predictive performance and limitations of EffHunter were compared with EffectorP 2.0 on noncanonical effectors (which are effectors hard to predict), such as SAD1, Mg3LysM, BEC1054, BEC1011, BEC1019, CSEP0055, Bcg1, CSEP0105, SIS1, Xyla, PIIN 08944, AvrPm3 and AvrSr35.

### 2.4. Prediction of Effector Proteins in Fungal Genomes

Deduced proteomes from 87 fungi and 4 oomycetes were downloaded from the databases of Broad Institute and Joint Genome Institute (https://jgi.doe.gov/) [42] and from the resource for genome-scale data, Ensembl Genomes (https://fungi.ensembl.org/index.html), developed by the EBI and the Welcome Trust Sanger Institute [43] (Supplementary Table S2).

## 3. Results

### 3.1. EffHunter: A Pipeline to Predict Fungal Effectors Proteins

The EffHunter pipeline was constructed for the in silico identification effectors in fungal proteomes. The architecture of EffHunter consists of four modules: (1) analysis of the protein length and cysteine count, (2) detection of signal peptide, (3) transmembrane domains and (4) subcellular localization. 

Length estimation and the counting of cysteines in each protein are performed by a set of Perl/Bioperl scripts on the subject proteins; the programs listed in Table 1 accomplished the other bioinformatics analysis.

**Figure 1:**
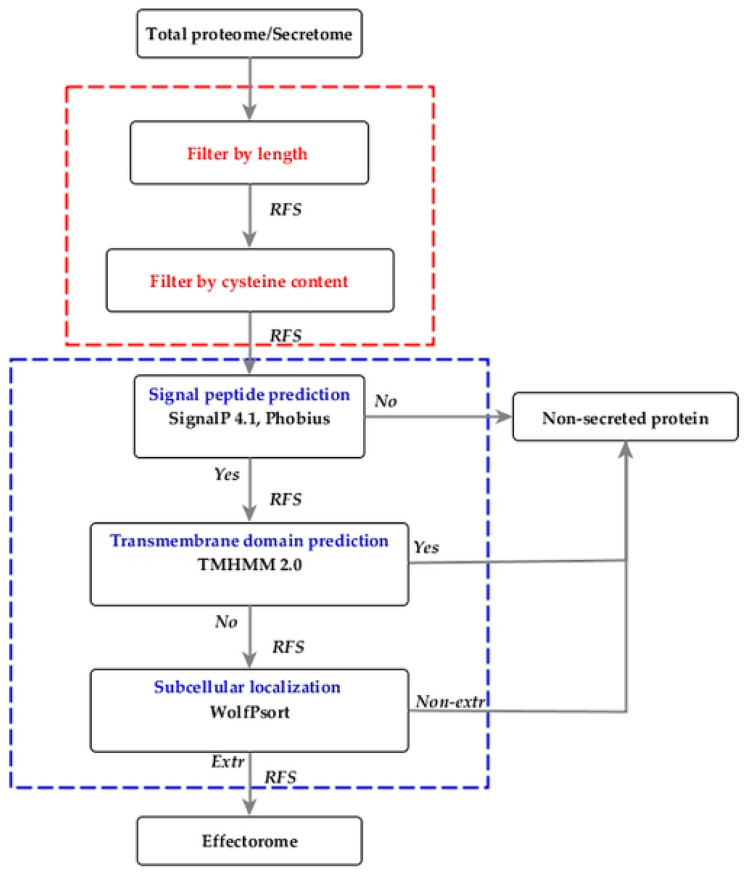
Complete EffHunter workflow for the prediction of effectors in fungal proteomes. Red square, custom tools (user parameters); blue square, pre-installed prediction tools. RFS, retrieved FASTA sequences. The pipeline works correctly either on total proteomes or secretomes.

The EffHunter pipeline compiles the SignalP 4.1, Phobius, TMHMM 2.0 and WoLFPSORT programs, together with a set of scripts in Perl, to execute the analyses of length and content of cysteine residues in a single step for each polypeptide sequence of fungal proteomes (Figure 1).

The analysis with the EffHunter pipeline involves the automatic sequential analysis of the FASTA file output from the previous module until the creation of the final output file.

### 3.2. Validation ab Initio

The evaluation of EffHunter’sperformance was done by employing the positive data set (Table 2), comprising 56 effectors available in the PHI-database and the 94 effectors used for the initial positive training of EffectorP 2.0 [15]. The final list comprises 150 protein effectors, which include 140 effectors from fungi and 10 protein effectors from oomycetes (Supplementary data set S1).

As negative controls, a subset of 4530 proteins was used, comprising P450 proteins, MFTS and ABC transporters, most of them large, integral membrane proteins. The set of negative controls comprises a large number of proteins to challenge EffHunter, in order to prevent false positive identification as much as possible. Using the same data set with positive and negative controls, we compared our pipeline with EffectorP 2.0, a machine learning classifier for fungal effector prediction.

EffHunter positively identified 105 from 150 effectors (70%); meanwhile, 45 were false negatives (30%). The missing 45 effectors could not be identified because some of them contain transmembrane domains or less than four cysteine residues, two criteria that EffHunter uses for the prediction. From the total subset of proteins evaluated (4680), no false positive was retrieved (Table 3). Sequential analyses with the programs and scripts that make up EffHunter produced the same results as the automatic analysis with EffHunter (Table 3), showing that the pipeline worked as expected.

EffectorP 2.0 identified 166 effectors; 102 of them were true effectors (68%), but 48 true effectors were missing, i.e., 32% of false negatives. The larger difference observed between both predictors was the number of false positives, e.g., 64 for EffectorP 2.0 (41 ABC transporters, 22 cytochrome P450, and 1 MFTS) and none for EffHunter. Furthermore, sensitivities are similar (76% and 75%), but specificity was larger for EffHunter (100%) in comparison with 98% for EffectorP 2.0. Precision of EffHunter was 100% vs. 97% for EffectorP 2.0. Accuracy value was 99% for EffHunter and 97% for EffectorP 2.0 (Table 3).

In addition, following the strategy of Sonah et al. [10], i.e., using the pipeline SECRETOOL and then selecting proteins with maximum length of 300 amino acids, 72 true effectors were recognized and 78 true effectors were discarded (false negatives). No false positive was retrieved in this prediction.

Estimation of the F1 score for these predictions was carried out considering their results on the same set of 4680 proteins (containing 150 true effectors and 4530 negative controls). The scores were 0.57 for EffectorP v1.0, 0.64 for EffectorP v2.0, and SECRETOOL/300 amino acids and finally, for EffHunter, 0.82. These results support EffHunter as a good predictor for fungal canonical effectors.

**Figure 2:**
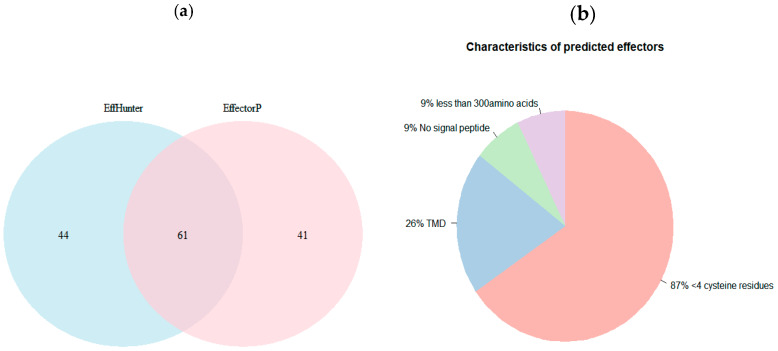
Effector prediction of positive control set. (**a**) Venn diagram showing the distribution of shared and non-shared predicted effectors by EffHunter and EffectorP 2.0. (**b**) Pie chart summarizing the characteristics of the 41 non-shared effectors protein predicted by EffectorP 2.0.

EffHunter and EffectorP 2.0 shared 61 candidates. Non-shared predicted candidates were 44 for EffHunter and 41 for EffectorP 2.0 (Figure 2a). All of the 44 EffHunter specific candidates meet the established criteria for effector prediction. From the 41 candidates predicted only by EffectorP 2.0, 9% have no signal peptide, 26% have TMDs and 87% have less than four cysteine residues (Figure 2b). Since effectors are so diverse, it is ambiguous how many of the specific candidates of each predictor are true effectors, but candidates of Effhunter highly meet its own established criteria. The algebraic sum in Figure 1 is greater than 100% because some candidates have two or more characteristics, e.g., no signal peptide and have TMD at the same time.

### 3.3. Validation of EffHunter with Fungal Proteomes and Comparison with Other Effector Prediction Tools

EffHunter is versatile and allows the user to set cutoff values for the protein sequence length and number of cysteine residues. To continue the validation of EffHunter in the prediction of effector proteins, additional analysis was carried out on a few economically important plant pathogens whose effectoromes have been previously analyzed. Parameters and software used in each case are described in Table 4. For EffHunter, the length of proteins was set according to the reported criterion in each case. The number of cysteines was fixed at ≥4 per protein since is difficult to set a proper percentage, as the number of cysteines varies with the size of the protein.

In the plant pathogen *Blumeria graminis* f. sp. *hordei*, EffHunter predicted 490 effector candidates in comparison with the 494 reported by Liang et al. [38] using diverse criteria presented in Table 4; 404 of them were common in both predictions. EffHunter identified 82 proteins which were not retrieved by the other study. These 82 proteins were then analyzed with different programs that are not included in EffHunter but were used by other authors (i.e., TargetP 1.1 [45] and big-PI [46]). In addition, the analysis of Liang et al. [38] used one additional criterion, searching for secreted proteins that show similarity only with proteins from powdery mildews. These analyses identified 12 false positives in EffHunter candidates because they did not meet this additional criterion, but 70 of them met all the author’s criteria. On the contrary, Liang et al. [38] identified 86 candidates, which were not recognized by EffHunter. Sixty-two of them were larger than 400 amino acids; 19 had no signal peptide and 5 were predicted as GPI-anchored. Therefore, these 86 candidates identified by Liang et al. [38] seem to be false positives according to their own criteria (Table 4).

The second comparison was with a list of candidate effectors reported by Chang et al. [39] from *Pseudocercospora fijiensis*, the causal agent of black Sigatoka disease in banana and plantain. Chang et al. [39] reported 105 candidate effectors for the fungus, while EffHunter predicted 136, with 78 of them shared between both analyses. From the 27 candidates exclusive of the results of Chang et al., 15 appear to be false positives since they do not meet some of their criteria; 12 were ambiguous. Ambiguity arises because both predictions have different settings for some criteria (i.e., they accepted one TMD in the proteins, but EffHunter does not). Since effectors are so diverse, the criterion of having one or no TMD has a similar probability of being acceptable. In the ambiguous candidates, seven have one TMD and five candidates have only two cysteines in their sequence, but they present the 2% cysteine because those are peptides with 60 amino acids or less. EffHunter parameters filtered those proteins. EffHunter predicted 58 effectors that were not enlisted in the reference data; 32 of them may be false negatives for the reference since they meet all their parameters. Sixteen EffHunter candidates were probably false positive, and 10 were ambiguous (Table 4).

The third comparison was with a list of deduced effectors from *Mycosphaerella graminicola*, a fungus causing septoria leaf blotch in wheat [40]. In addition to some software previously mentioned (i.e., SignalP 3.0, TargetP 1.1, big-PI, WoLFPSORT), these authors included in their analysis the use of LocDB [47], ProtComp v8.0 and PotLocDB [48] and designed a script to set cysteine at 5%. The list of candidates was filtered to exclude those proteins that have any functional annotation. They reported 171 effector candidates vs. 183 by EffHunter.

One hundred ten were common in both results; among the 61 candidates exclusive for the reference, 60 failed to meet some of the authors’ criteria (they may represent false positives), but one was true positive for their result and false negative for EffHunter. Curiously, this protein (ID 82029) is not present in the nonredundant set of *M. graminicola* proteins in the JGI database. For this reason, EffHunter did not analyze it.

EffHunter predicted 74 candidates in addition to the common set. Fifty of these candidates are true positives, according to all criteria from authors, including the search for candidates with no relation with proteins with functional annotation. These 50 candidates are true effector candidates for EffHunter and false negatives for the reference. It is probable that EffHunter had 24 false positives since four have GPI, ProtComp predicts seven non-extracellular proteins, and 13 have homology with proteins with functional annotation in Pfam. EffHunter does not include the search of GPI, the ProtComp program and the Pfam database in the pipeline.

In summary, EffHunter performed well in these comparisons. In the three cases, the number of true positives was higher, and the false positives were lower with EffHunter. The number of false negatives for EffHunter was negligible; meanwhile, in the other reports, it was 14% in the Liang et al. [38] effectorome prediction for *B. graminis* f. sp. *hordei*, 30% in Chang et al. [39] prediction for *P. fijiensis* and 29% in Morais do Amaral et al. [40] prediction for *M. graminicola*, supporting the robust and reliable prediction by EffHunter.

In another report, Sonah et al. [10] used the bioinformatics tool SECRETOOL on 12 proteomes [41] to obtain the respective secretomes and then retrieve the small proteins (≤300 amino acids), proteins that they classified as effectors (Table 5). One of these pathogens (*M. graminicola*) was analyzed also by Morais do Amaral et al. [40] and compared here with EffHunter (Table 4), which is interesting because this expands the comparison among the different predictive tools for fungal effectors. Comparison among effectoromes predicted by SECRETOOL, EffHunter and EffectorP 2.0 was conducted for these pathogens (Table 5). Sonah et al. [10] did not provide a link to the databases that they used; as such, the first attempt in our analyses was conducted on total proteomes of these species. EffHunter works properly on total proteomes, but we observed an elevated number of false positives with EffectorP 2.0. Using a total proteome as input, EffectorP 2.0 identified 1663 candidates for *C. fulvum* vs. 151 candidates with a secretome as input (data not shown). For this reason, the secretome for each pathogen was first obtained submitting the proteomes to the SignalP 4.1to reduce the number of false positives for the EffectorP 2.0 analysis. Table 5 shows the prediction of effectors by the three programs. The numbers of effectors are similar between Sonah et al. [10] predictions and EffHunter; in general, EffectorP 2.0 predicted in some cases a lower number of candidates than EffHunter and SECRETOOL [10].

For example, in the case of *Puccinia graminis* f. sp. *tritici*, 659 effector candidates were predicted by EffHunter, 612 reported by SECRETOOL and 605 predicted by EffectorP 2.0. In the latter, 110 proteins have one or more transmembrane domains and cannot be classified as positive or negative, but as ambiguous. In the list of 94 validated effectors shown in Table 2 [15], 11 have one TMD and 83 have no TMD. In general, in each effectorome, EffectorP 2.0 retrieved few candidates with no signal peptide or possessing transmembrane domains. False positives or false negatives from Sonah et al. [10] could not be calculated because of the lack of the datasets of their sequences. Step-by-step analyses of all candidates retrieved by EffHunter showed that they meet all EffHunter criteria, suggesting no false positives in our sets of putative effectors.

To further evaluate EffHunter, its performance on nonconventional known effectors of five species of phytopathogens was analyzed and compared with EffectorP 2.0 (Table 6). PIIN 08944 and AvrSr355 are elusive effectors, and neither EffHunter nor EffectorP 2.0 can recognize them. SAD1 and BEC1054 are not recognized by EffHunter, but they are predicted as effectors by EffectorP 2.0. On the contrary, EffHunter recognizes Mg3LysM, BEC1019 and CSEP0105, which are not recognized by EffectorP 2.0. These results show that EffHunter has strengths and limitations, as does EffectorP 2.0, which is currently the predictive tool for fungal effectors most widely used in the literature. Even with this limitation, EffHunter’s performance is acceptable to search for effectors in fungal proteomes and has the characteristic of being able to perform the analyses on total proteomes without prior filtering of the protein set to retrieve the secretome. The results show that EffHunter is a tool that makes the search for effectors friendly, making it a better tool.

### 3.4. Prediction of Effector Proteins in Several Fungal and Oomycetes Proteomes with EffHunter

The prediction of candidate effectors with EffHunter was carried out on 95 proteomes downloaded from the JGI Genome Institute Mycocosm and FungiEsembl platform (Supplementary Table S2).

Since effectors have been defined as pathogenicity-related proteins, effectoromes were compared among 40 phytopathogens with different lifestyles: 9 species of biotrophs (blue bars), 20 species of hemibiotrophs (green bars) and 11 species of nectrotrophs (red bars). In general, the highest number of predicted effectors was in the group of hemibiotrophs (close to 400 on average), with the lower number in the necrotrophs (around 200 effectors), followed the biotrophic group (around 300 effectors). In each group, there are fungi with an expanded or contracted set of effectors, such as the necrotroph *Penicillum digitatum,* the hemibiotrophs *Verticillium dahliae* and the oomycete *Phytophthora capsici*, and the biotrophs *Blumeria graminis* f. sp. *tritici* and *Ustilago maydis,* with smaller effectoromes than the rest.

**Figure 3:**
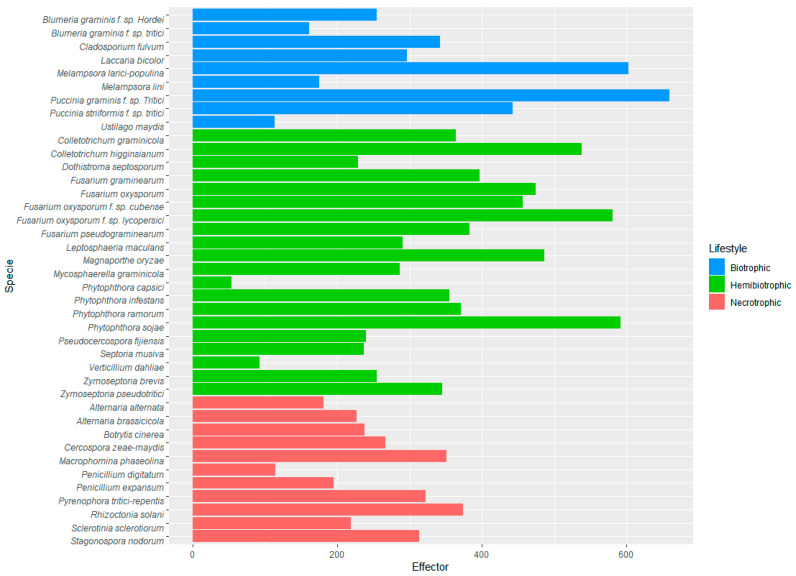
Effectoromes in fungal and oomycetes phytopathogens predicted by EffHunter.

The performance of EffHunter predicts expanded effectoromes in *Melampsora larici-populina*, *Puccinia graminis* f. sp. *tritici*, *Colletotrichum higginsianum*, *Fusarium oxysporum* f. sp. *lycopersici*, *Magnaporte oryzae* and *Phytophthora sojae* (Figure 3 and Supplementary Table S3).

In Figure 3, the graphic shows the number of effectors predicted in different types of fungi: yeast, brown and white rot fungi, ectomycorrhiza, opportunistic, mycoparasites, human pathogens, plant pathogens, entomopathogens and saprotrophs (Supplementary Table S4).

The lowest numbers of effectors were predicted in yeast. Curiously, the number of effectors in human pathogens was lower than in the nonpathogenic group, such as ectomycorrhiza and saprotrophs, revealing that pathogens have diversity in the number of predicted effectors. The number of effectors predicted in other pathogens was congruently larger. The groups of fungi with more effectors were plant pathogens and entomopathogens. However, it is not exclusive for pathogens to have the largest effectoromes, because in the case of saprotrophs, EffHunter predicted a similar number of effectors than in phytopathogens and entomopathogens.

## 4. Discussion

Effectors are key molecules in pathology since they enable the pathogen to modify host cell structure, physiology and metabolism to permit pathogen growth and colonization in the host. Most of the available knowledge has emerged from the study of plant–pathogen interactions where it has been discovered that effectors play diverse functions. Many effectors work as plant immunosuppressors, but others can trigger sugar transport in benefit of the pathogen [60], block or inactivate plant receptors preventing its detection [61] or, on the contrary, activate the immune plant receptors to kill the host [62]. Research on effectors is at its peak, and surely many other roles of effectors remain to be discovered. 

In agriculture, fungal diseases cause major losses in several high-value crops such as bananas, coffee, cacao, legumes and spices, provoking ~20% of annual crop yield losses worldwide [63]. Effector proteins are potential biotechnological tools to assist in developing disease control strategies, for example, to select effector-assisted tolerant or resistant plants in breeding programs [64] or to identify plant targets and protein cognates for genetic engineering [65].

High-throughput technologies can currently provide us with high-quality transcriptomes from plant–pathogen interaction and pathogen genomes, meaning powerful data output for effector discovery. However, although progress has been achieved in functional validation of effectors, and multiple candidates can be tested [66], the experimental validation requires experience in genetic engineering. Moreover, this validation is expensive and time-consuming. Currently, less than 200 effectors have been identified and characterized [15]. Hence, robust effector mining from genomic data is key.

EffHunter is a pipeline created in our group by integrating SignalP 4.1, Phobius, WoLFPSORT, TMHMM 2.0 and two scripts for filtering small size (≤400 amino acids) and cysteine-rich (≥ 4) proteins. EffHunter identifies canonical effectors, i.e., secreted, apoplastic, small size and cysteine-rich. To test EffHunter, we used a positive subset of 94 effectors for the initial positive training of EffectorP 2.0 [15] and 56 effectors available in the PHI-database. A large set of negative controls (4530 proteins) was used to challenge EffHunter. No protein in the negative control set is extracellular and, more importantly, none has been described as a fungal effector. In addition, they are highly variable in length, content of cysteine, TMDs and presence or absence of signal peptide, in order to prevent false positive identification by EffHunter as much as possible. 

Performance of EffHunter was compared with other effector predictors, running the analysis on the same set of data (4680 proteins, comprising 150 true effectors and 4530 negative controls). F1 score for EffHunter was 0.82, superior to F1 scores for EffectorP v1.0 and effector prediction following the strategy of Sonah et al. [10] (Table 3). EffectorP v2.0 showed a lower F1 score than EffHunter (0.64). The larger difference observed between EffHunter and EffectorP v2.0 was the number of false positives, 64 for EffectorP 2.0 and none for EffHunter. 

The positive set of proteins used for developing and training EffectorP 2.0 comprised effectors with transmembrane domains (11 proteins), effectors with no signal peptide (2 proteins), effectors larger than 400 amino acids (2 proteins) and 37 proteins with less than four cysteine residues [15]. This training enables that algorithm to identify effectors with these noncanonical characteristics; however, according to our analysis, it also results in a higher percentage of putative false positives, in contrast to the zero false positives obtained with EffHunter. Another advantage of EffHunter is its suitability for total proteomes or secretomes as input, retrieving the same number of candidate effectors, while EffectorP 2.0 requires a secretome as input. We found that effector prediction using a total proteome in EffectorP 2.0 increases the rate of false positives by almost 10 times (data not shown). EffHunter demonstrated a very good performance as it relates to sensitivity, specificity, precision and accuracy, which were similar to or better than those of EffectorP 2.0.

The ability to predict fungal effectors by the EffHunter pipeline was compared later with reports that used different strategies to predict effectors in *Blumeria graminis* f. sp. *hordei* [38], *Pseudocercospora fijiensis* [39] and *M. graminicola* [40]. We found that similar numbers of effectors were predicted between EffHunter and each of these reports where distinct strategies and criteria were used. For instance, to predict effectors of the different fungi with EffHunter, the length of amino acids was set according to each report. The number of cysteines was not changed because, in the case of the report for *B. graminis* f. sp. *hordei*, the number of cysteines was not defined, and in the case of *Pseudocercospora fijiensis* and *Mycosphaerella graminicola,* authors used, respectively, 2% and 5% cysteine as cut off. However, 2% and 5% would discard many promising candidates. Then, since both extremes are not adequate, the number of cysteines for EffHunter searching was set at ≥4 as used in other analyses in this manuscript, reinforcing the EffHunter evaluation against other analyses, which use different parameters.

EffHunter performed well on each comparison. In the three cases, their number of true positives was higher and the number of false positives (candidates that do not meet the criteria established by the respective authors) was lower in comparison with these reference works. EffHunter false positives resulted from additional criteria used by the authors, for example, discarding candidates that have homologs in fungi phylogenetically distant from the model under study or that exclude those that have homologous proteins with any functional annotation. These criteria are good, but we do not recommend including these criteria in automatic analysis, to prevent elimination of many potential true effectors since 18% of true effectors have functional annotation (e.g., hydrophobin, protein with CFEM-domain, cerato-platanin, etc). In the case of false negatives (those candidates proposed only by the other predictor, which largely qualify as potential effectors), the number was negligible for EffHunter; meanwhile, it was 70 in the Liang et al. [38] effectorome prediction for *B. graminis* f. sp. *hordei*, 32 for Chang et al. [39] for *P. fijiensis* and 50 for Morais do Amaral et al. [40] prediction for *M. graminicola*. Such diversity of approaches used with other fungi presented a great challenge to EffHunter; however, we demonstrated its capacity to perform smoothly with different data.

Another advantage of the program is its versatility since the user can set the cut-off for the length (number of amino acids) and the number of cysteines.

The next evaluation was to compare with the report from Sonah et al. [10] since these authors used another bioinformatics tool, SECRETOOL [41] to predict effectoromes in proteomes of 12 fungi; they filtered first by the SECRETOOL pipeline and then selected the proteins ≤ 300 amino acids. The number of effectors predicted by EffHunter was consistent with the number of predicted effectors by the SECRETOOL pipeline used by these authors, probably because both predictors share similarities in their constructions (both comprise analyses by SignalP 4.1, TMHMM 2.0 and WoLFPSORT). However, they are not identical, since SECRETOOL does not analyze the content of cysteine and EffHunter does not integrate TargetP 1.1 and PredGPI as SECRETOOL does. Results obtained with both tools were not similar for all organisms, revealing differences between both predictors. Unfortunately, coincidences, differences, false positives and false negatives in the predictors for both bioinformatics tools could not be checked, because Sonah and colleagues did not provide the sequences or ID of their effector proteins. In the case of the candidates predicted by EffHunter, all of them met EffHunter’s criteria, reinforcing its high accuracy and its low false positive rate. EffectorP 2.0 predicted a lower number of effectors in all these cases.

Altogether, EffHunter demonstrated that it is a highly efficient bioinformatics tool for fungal effector prediction, and it can be a suitable tool to search effectoromes in fungal proteomes.

**Figure 4:**
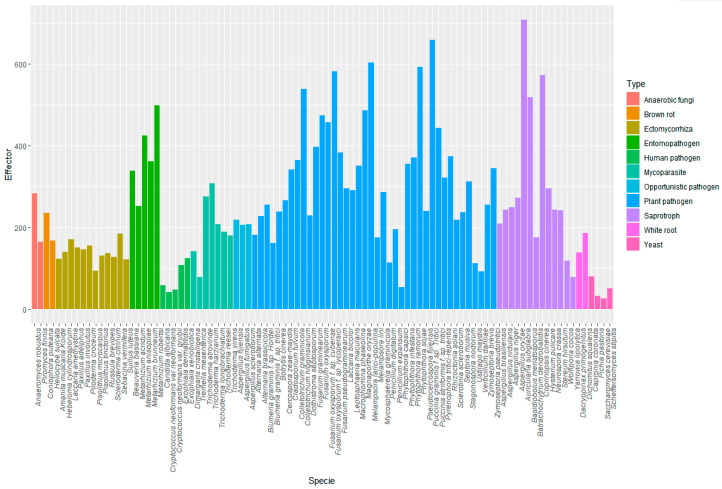
Predicted effectors in fungal and oomycetes proteomes using EffHunter.

Then, EffHunter was used to predict effectors on different types of fungi. The lowest numbers of effectors were predicted in yeast (Figure 4), consistent with what was reported by Sperschneider et al. [16]. These authors proposed that nonpathogenic fungi have less effectors than ectomycorrhiza and saprotrophs. EffHunter predicted a similar number of effectors in ectomycorrhizal as in white and brown rots. In congruence with these findings, recent literature evidences that small-secreted effectors participate in all types of microbial interactions, and the concept “effector” seems to be rapidly evolving [67,68,69,70,71,72].

In plant–pathogen interactions, effectors can be recognized by the cognate R proteins and trigger a hypersensitive response to prevent the spread of the pathogens. Some virulence factors are shared between plant and human fungal pathogens [73], but mammals have authentic immune systems, and their interactions with pathogens are different in comparison with plants. It is likely that some effectors from animal pathogens have different characteristics than phytopathogen effectors, and as a result, EffHunter could not identify them.

The highest numbers of effectors were predicted for plant pathogens and entomopathogens, suggesting that interactions with host producing toxic, antifungal metabolites demand a larger inventory of effectors. Consistent with this interpretation, *Metharrhizium anisoplae*, a generalist entomopathogen, has 68% more effectors than the specialist *Metarrhizium acridum*, enabling *M. anisoplae* to face more divergent challenges. The next group according to the number of effectors was the saprotrophic fungi. Effectors in saprotrophs are probably used for antagonism or in the interaction with microorganisms which inhabit decaying wood. Another explanation is that saprotrophs have effectors because when circumstances change, they could become pathogens [74,75].

Although fungi with small genomes (i.e., yeast) have a smaller number of effectors (30-50 effectors), and the size of effectoromes is not related with the size of the genome. For instance, *Puccinia graminis* f. sp. *tritici* (88Mbp) has 659 effectors, *Blumeria graminis* f. sp. *tritici* (158 Mpb) has 161 effectors, *Magnaporthe oryzae* (41 MbP) has 486 effectors and *Melampsora lini* (189 Mbp) has 175 effectors. 

The sizes of effectoromes seem to be related with lifestyles of the fungi: the lower number of effectors was observed in necrotrophs (average ~200 effectors). More complex interactions of biotrophs and hemibiotrophs require larger effectoromes (~300 and ~400 effectors, respectively). Evasion of host perception, suppressing host defense responses and keeping the host alive demand large catalogs of effectors in biotrophic and hemibiotrophic fungi.

As mentioned above, some fungi have unusual large effectoromes (600-700 effectors). The largest effectoromes were predicted for *Auricularia subgrabra* (708), *Puccinia graminis* f. sp. *tritici* (659) and *Melampsora larici-populina* (603) (Supplementary Table S2). Largest sets of effector candidates in *Puccinia graminis* f.sp. *tritici* and *Melampsora larici-populina* are consistent with predictions by different programs, reported by Sperschneider et al. [15]; these authors proposed that these large effectoromes exist because these pathogens require two host species to complete their cycle of life. Recently, Liang et al. [38] investigated evolutionary features of the genes in obligate biotrophic fungal pathogens and reported that secreted effectors in powdery mildews of monocots have been subjected to positive selection, which explains the expansion of effectoromes in *P. graminis* f.sp. *tritici* and *M. larici-populina.* On the contrary, the families of secreted effectors in powdery mildews of dicots have been under strong purifying selection, resulting in the contraction in the number of effectors, e.g., *Melampsora lini* (175 effectors). The number of effectors in *Auricularia subgrabra* is large, probably also by expansion of the family of secreted proteins.

On the other hand, it is known that the characteristics of the fungi and oomycete effectors are different, but we took advantage of the fact that both of their effectors have signal peptides for secretion, and we used EffHunter to predict effectors in oomycetes. The number of candidates predicted by EffHunter in *P. infestans* (355) was similar to the prediction reported by Sonah et al. [10] (343 candidates). Supporting EffHunter’s prediction, 295 candidates contain the motif RxLR, very common in oomycete effectors. This suggests that EffHunter is suitable for searching effectors in oomycete proteomes. Haas et al. [57], using Hidden Markov Models to retrieve proteins with oomycete motifs, predicted 563 effectors in *P. infestans*. Restriction by protein length and cysteine content by EffHunter can underestimate the number of effectors in oomycetes, but this pipeline can be used for easy and rapid preliminary searches. 

It is important to highlight that any of the available effector predictors is capable to identify all effectors that have been experimentally studied so far [15]. Effector BEC1019, a haustorial protease from *Blumeria graminis* f. sp. *hordei* that suppresses host cell death, and AvrSr35, a 578 amino acids effector from *Puccinia graminis* f. sp. *tritici,* are neither identified by any effector classifier previously created, nor by EffHunter. EffectorP 2.0 does not retrieve the effectors Mg3LysM from *Zymoseptoria tritici* and CSEP0105 from *Bumeria graminis f.sp. hordei;* meanwhile, both of them are recognized by EffHunter. On the contrary, BEC1054 and BEC1011 from *Blumeria graminis* f. sp. *hordei* are identified by EffectorP 2.0, but EffHunter is not able to recognize them as effectors. A combination of different tools can increase sensitivity in effector prediction, adding the criteria and the predictive advantages of each tool. For example, a combination of EffectorP 1.0 and EffectorP 2.0 allowed the identification of AvrSr50 effector from *Puccinia graminis* f. sp. *tritici* [15]. However, larger lists make it more difficult to prioritize candidates for functional validation. The main robustness of EffHunter is its low false positive rate in identifying bona fide canonical effectors. Although EffHunter ignores effectors with atypical characteristics, its high PPV (100%) and accuracy (ACC) (99%) make it a useful tool for the selection of top candidates. This is crucial because the number of fungal effectors per genome is in the order of hundreds [10,16] and false positives slow down the validation and characterization of effectors. After effector mining, high-priority candidates can be selected by filtering with additional criteria (when available) common among many known effectors such as in planta expression data, genomic location (e.g., comprising clusters of putative effectors, or locations in dispensable chromosomes), positive net charge and low content of serine and tryptophan, among others.

## 5. Conclusions

EffHunter is a pipeline that integrates the software SignalP 4.1, Phobius, TMHMM 2.0 and WoLFPSORT with Perl scripts to filter proteins by length and by cysteine content to search for fungal protein effectors in a single step. This makes EffHunter a user-friendly and amenable tool.

EffHunter is a robust tool that can identify effectors in fungal proteomes, showing higher accuracy and lower false positives than other effector predictors do.

Different types of fungi have varying quantities of effectors. Although exceptions were observed, there are averages in the number of effectors in each type of fungi. The results of our effectoromics study showed that plant pathogens and entomopathogens were the organisms with the largest effectoromes. Within plant pathogens, as it relates to their lifestyle, biotrophic and hemibiotrophic fungi have larger effectoromes than necrotrophic fungi.

## 6. Patents

The present pipeline was certified at Mexican Public Copyright Registry with the registration number 03-2019-101809310300-01.

## Figures and Tables

**Table 1:** Bioinformatics tools integrated into EffHunter.

Program	Features	Website	Reference
Perl/Bioperl	International association of users and developers of open-source Perl tools for bioinformatics, genomics and life science.	https://bioperl.org/	[44]
SignalP 4.1	Predicts the presence of signal peptides and the location of their cleavage sites in proteins from gram-positive bacteria, gram-negative bacteria and eukarya.	http://www.cbs.dtu.dk/services/SignalP/index.php	[12]
Phobius	This server is for the prediction of transmembrane topology and signal peptides from the amino acid sequence of a protein.	http://phobius.sbc.su.se/	[18]
WoLFPSORT	Converts protein amino acid sequences into numerical localization features, based on sorting signals, amino acid composition and functional motifs, to predict protein subcellular location.	https://wolfpsort.hgc.jp/	[13]
TMHMM 2.0	Predicts trans-membrane (TM) domain helices in proteins.	http://www.cbs.dtu.dk/services/TMHMM/	[14]

**Table 2:** Positive data set of effector proteins used in this work.

Species	Effector Proteins
*Alternaria alternata*	**Aapg1**
*Alternaria citri*	**Acpg1**
*Aspergillus flavus*	**PECA**
*Aspergillus fumigatus*	**CfmB, CfmC**
*Beauveria bassiana*	**BbCHIT1**
*Bipolaris maydis*	**Ppt1**
*Bipolaris oryzae*	**Ppt1**
*Bipolaris zeicola*	**PGN1**
*Blumeria graminis* f. sp. *hordei*	Avrk1, Avra1, Avra13, **CSEP0105, BEC1005, BEC1040**
*Blumeria graminis* f. sp. *tritici*	AvrPm2
*Botrytis cinerea*	**CUTA**, Nep1, **PGIP2, BcPGA1, BcPG2**
*Botrytis elliptica*	**BeNEP2**
*Candida albicans*	**BGL2, SAP1, sap1, SAP2, SAP3, sap3, Pga26, RBT4**
*Candida tropicalis*	**ACP**
*Cladosporium fulvum*	Avr2, Avr4, Avr4E, Avr5, Avr9, Ecp1, Ecp2, Ecp4, Ecp5, Ecp6
*Claviceps purpurea*	**CPPG1, CPPG2**
*Colletotrichum graminicola*	CgEP1, Cgfl
*Fusarium graminearum*	FGL1
*Fusarium oxysporum*	**Avr3, PG1, XYL3**
*Fusarium oxysporum* f. sp. *lycopersici*	Six1, Six2, Six3, Six4, Six5, Six6, Six7, Six8
*Fusarium solani*	**cutA**
*Histoplasma capsulatum*	**CBP1**
*Laccaria bicolor*	MiSSP7
*Leptosphaeria maculans*	AvrLm1, AvrLm4–7, AvrLm6, AvrLm11, **AvrLmJ1, SP1**
*Magnaporthe oryzae*	Avr1-CO39, Avr-Pia, AvrPib, Avr-Pita, Avr-Pii, Avr-Pik, AvrPi9, AvrPiz-t, Bas1, Bas2, Bas3, Bas4, Bas107, Bas162, **GAS1**, **GAS2**, Iug6, Iug9, MC69, **MHP1**, MoCDIP1, MoCDIP2, MoCDIP3, MoCDIP4, MoCDIP5, **MPG1**, MoHEG13, Msp1, Pwl1, SPD2, SPD4, SPD7, SPD9, SPD10, **XYL-6**
*Melampsora lini*	AvrL2-A, AvrL567-A, AvrM, AvrM14, AvrP4, AvrP123,
*Metarhizium anisopliae*	**Pr1**
*Monilinia fructicola*	**MfCUT1**
*Parastagonospora nodorum*	**SP1**
*Phakopsora pachyrhizi*	PpEC23
*Phytophthora cactorum*	**PcF**
*Phytophthora capcisi*	**Pc129892, Pcipg2**
*Phytophthora infestans*	**EPI10, INF1, INF2A, INF2B**
*Phytophthora parasitica*	**CBEL, Ppxyn1**
*Phytophthora sojae*	**GIP2**
*Puccinia graminis* f. sp. *tritici*	AvrSr50, PGTAUSPE-10-1
*Puccinia striiformis* f. sp. *tritici*	Pec6, PstSCR1
*Pyrenophora tritici-repentis*	ToxB
*Rhynchosporium secalis*	NIP1, NIP2, NIP3
*Sclerotinia sclerotiorum*	SsSSVP1
*Stagonospora nodorum*	ToxA, Tox1, Tox3
*Trichoderma virens*	**Sm1**
*Uromyces fabae*	RTP1
*Ustilago hordei*	UhAvr1
*Ustilago maydis*	Cmu1, eff1-1, **Mig1**, **Mig2-1**, Pep1, Pit2, Tin2, See1
*Verticillium dahliae*	Ave1, PevD1, Vdlsc1, VdSCP7
*Zymoseptoria tritici*	AvrStb6, Zt6

* One hundred fifty effector proteins were pooled from the list (94) collected by Sperschneider et al. [15] and 56 effectors retrieved from the PHI database (labeled in bold). All these effector proteins have been published in peer-reviewed journals.

**Table 3:** Validation of EffHunter for prediction of effector proteins and comparison with EffectorP 2.0.

**Step-by-Step Prediction**														
Data*	Proteins in Data set	Total Proteins	Length (30—400aas)	>4 Cysteine	Signal peptide by SignalP/Phobius	Proteins without TMD with TMHMM	Total prediction	Results	Sen/Rec	Spe	PPV/Prec	ACC	FPR	F1 score
Set 1	150	4680	765	435	107	105	105	105	70%	100%	100%	99%	0.00%	0.82
Set 2	2329							0						
Set 3	476							0						
Set 4	1725							0						
**EffHunter**														
Data*	Proteins in data set	Total proteins		Total prediction		Prediction		Sen/Rec		Spe	PPV/Prec	ACC	FPR	F1 score
Set 1	150	4680		105		105		70%		100%	100%	99%	0.00%	0.82
Set 2	2329					0								
Set 3	476					0								
Set 4	1725					0								
**EffectorP 2.0**														
Data*	Proteins in data set	Total proteins		Total prediction		Prediction		Sen/Rec		Spe	PPV/Prec	ACC	FPR	F1 score
Set 1	150	4680		166		102		68%		98%	61%	97%	1.41%	0.64
Set 2	2329					41								
Set 3	476					22								
Set 4	1725					1								
**EffectorP 1.0**														
Data*	Proteins in data set	Total proteins		Total prediction		Prediction		Sen/Rec		Spe	PPV/Prec	ACC	FPR	F1 score
Set 1	150	4680		164		91		60%		98%	55%	97%	1.6%	0.57
Set 2	2329					49								
Set 3	476					20								
Set 4	1725					4								
**Sonah et al.** [10] **(SECRETOOL and filter by length <300 amino acids)**														
Data*	Proteins in data set	Total proteins		Total prediction		Prediction		Sen/Rec		Spe	PPV/Prec	ACC	FPR	F1 score
Set 1	150	4680		72		72		48%		100%	100%	98%	0.00%	0.64
Set 2	2329					0								
Set 3	476					0								
Set 4	1725					0								

* Set 1: Positive dataset of true effectors (positive dataset comprises effectors retrieved from pathogen-host interaction (PHI) and the list collected by Sperschneider et al. [15]); Set 2: ABC transporters; Set 3: cytochrome P450; Set 4: proteins classified as major facilitator transporter superfamily. Sen/Rec: Sensitivity/Recall; Spe: Specificity; PPV/Prec: Positive Predictive Value/Precision; ACC: Accuracy; FPR: False positive rate; F1 score: Measure of the success of binary classifier (score reaches its best value at 1, and worst score at 0).

**Table 4:** Data analysis of the set of fungal effector proteins between EffHunter and other reports.

Species	Criteria in Reference	Genome Size (Mbp)	Total Proteome	Secretome	Effectors Prediction in Reference	EffHunter Prediction	Shared	Difference in: Reference(R) EffHunter(E)	Observations for Effectors Predicted by Reference or by EffHunter	Summary of True or False Positives or Negatives, or Ambiguous in the Specific Sets of Effectors (Considering Both Predictions)
*Blumeria graminis* f. sp. *hordei* [38]	Secretion signal with SignalP 4.1 and SecretomeP, no TMD with TMHMM 2.0, no hits outside powdery mildews with Blastp; subcellular localization with TargetP 1.1 and GPI anchors by Big-PI.	124.49	7118	726	494	490	408	R = 86	**Negatives (86): 62** proteins are larger than 400 amino acids, **5** proteins have GPI binding and **19** proteins have no signal peptide and have TMD. Therefore, all 86 are false positive in this set. TP = 0; FP = 86	Reference TP = 0 FP = 86 FN = 70 TN = 12
								E = 82	**Negatives (12): 3** proteins were predicted by TargetP 1.1 as mitochondria target, **6** proteins were predicted with GPI anchors by big-PI and **3** proteins have homologs in fungal species that are not powdery mildew. **Positives** (**70**): **70** meet all criteria of authors; Blastp retrieved homologs only in powdery mildews. **TP = 70; FP = 12**	EffHunter TP = 70 FP = 12 FN = 0 TN = 86
*Pseudocercospora fijiensis* [39]	Secretion signal with SignalP 4.1, one or no TM domains with TMHMM 2.0, subcellular localization with TargetP 1.1 and WoLFPSORT, no GPI anchor with PredGPI, length <250aas, >2% cysteine residues	74.1	13,107	584	105	136	78	R = 27	**Negatives (15)**: **2** proteins are larger than 250 amino acids, **2** have no clear localization prediction with TargetP 1.1, **7** have no signal peptide, **4** have no extracellular location. *Ambiguous (12): **7** have one TMD and **5** small proteins (<60 amino acids) with less than 4 cysteine, but ≥2% cysteine. **A = 12; FP = 15**	Reference TP = 0 FP = 15 FN = 32 TN = 16 A = 12
								E = 58	**Negatives (16): 11** proteins are GPI-anchored, **5** proteins have mitochondria target (by TargetP 1.1). **Positives (32): 30** meet all criteria and have 2.0-8.2% cysteine; **2** proteins are predicted with signal peptide by SignalP 4.1 and Phobius, and as extracellular with WoLFPSORT, but TargetP 1.1 (reference) fails to predict localization. *Ambiguous (10): **10** are >200 amino acids and have 4 (but <2%) cysteine. Meet all other criteria. **TP = 32; FP = 16; A = 10**	EffHunter TP = 32 FP = 16 FN = 0 TN = 15 A = 10
*Mycosphaerella graminicola* [40]	Size <200 amino acids; secretion signal with SignalP 4.1, one or no TM domain with TMHMM 2.0, secreted by TargetP 1.1, no GPI-anchor with big-PI, subcellular localization with WoLFPSORT and ProtComp, and no functional information	39.7	10933	492	171	183	110	R = 61	**Negatives (60): 37** proteins are larger than 200 amino acids; **3** do not have signal peptide; **6** are GPI-anchored, **14** are not predicted as secreted by ProtComp. **Positives (1):** Protein ID 82029 matches all criteria. This protein is not in the nonredundant set of *M. graminicola* database at JGI, therefore EffHunter could not analyze it. **TP = 1; FP = 60**	Reference TP = 1 FP = 60 FN=50 TN=24
								E = 74	**Negatives (24): 4** proteins have GPI anchors; **1** has mitochondria target; **6** are predicted cytosolic, mitochondrial or nuclear by ProtComp and **13** proteins have functional annotation in PFAM **Positives (50): 50** match all criteria and have no functional annotation or known protein domains. **TP = 50; FP = 24**	EffHunter TP = 50 FP = 24 FN=1 TN=60

* Ambiguous: Those candidates that meet criteria from one prediction (positive for this analysis), but do not meet criteria of the other analysis and criteria from one or the other are not definitive for assigning them as positive or negative. Databases analyzed in the references and by EffHunter were the same, except for *M. graminicola*. The authors did not provide that database; the nonredundant protein models from *M. graminicola* at JGI were downloaded in that case.

**Table 5:** Effector prediction data from the different predictions tool across 12 proteomes.

Species	Lifestyle	Genome		Total Proteins	Effector Predictions			Effectors in Reference	Reference Genome
		Mb	Coverage		EffHunter	EffectorP 2.0	*SECRETOOL		
*Alternaria brassicicola*	Necrotroph	31.03	120×	10688	227	113	228	139	[49]
*Blumeria graminis*	Biotroph	158.94	13×	6526	255	109	143	437	[50]
*Cladosporium fulvum*	Biotroph	61.11	21×	14127	342	151	296	271	[51]
*Colletotrichum graminicola*	Hemibiotroph	51.6	9×	12006	364	159	352	177	[52]
*Fusarium oxysporum*	Hemibiotroph	55.72	186.1×	17726	474	256	361	364	[53]
*Leptosphaeria maculans*	Hemibiotroph	44.81	8.31×	12469	290	162	263	529	[54]
*Magnaporthe oryzae*	Hemibiotroph	41.7	7×	12755	273	368	528	163	[55]
*Mycosphaerella graminicola*	Hemibiotroph	39.7	8.9×	10933	286	166	235	NS	[56]
*Phytophtora infestans*	Hemibiotroph	228.54	7.6×	17787	355	404	343	563	[57]
*Puccinia graminis* f. sp. *tritici*	Biotroph	88.64	6.9×	15979	659	605	612	1106	[26]
*Pyrenophora tritici-repentis*	Necrotroph	37.84	98×	12169	322	182	328	317	[58]
*Ustilago maydis*	Biotroph	19.66	10×	6785	113	107	142	426	[59]

* Prediction in Sonah et al. [10]; NS: Not specified

**Table 6:** Comparison of prediction between EffHunter and EffectorP 2.0 on noncanonical effectors.

Species	Effector	Length	No. of Cysteine	Signal Peptide	*TMD	EffHunter	EffectorP 2.0
*Sporisorium reilianum*	SAD1	626	4	No	0	Non-effector	Effector
*Zymoseptoria tritici*	Mg3LysM	232	9	Yes	0	Effector	Non-effector
*Blumeria graminis* f. sp. *hordei*	BEC1054	118	2	Yes	0	Non-effector	Effector
	BEC1011	118	3	Yes	0	Non-effector	Effector
	BEC1019	316	8	Yes	0	Effector	Non-effector
	CSEP0055	122	3	Yes	0	Non-effector	Effector
	Bcg1	146	2	Yes	0	Non-effector	Effector
	CSEP0105	128	6	Yes	0	Effector	Non-effector
*Rhizophagus irregularis*	SIS1	149	2	Yes	1	Non-effector	Effector
*Fusarium graminearum*	Xyla	231	1	Yes	0	Non-effector	Effector
*Piriformospora indica*	PIIN 08944	120	0	Yes	0	Non-effector	Non-effector
*Blumeria graminis* f. sp. *tritici*	AvrPm3	130	2	Yes	0	Non-effector	Effector
*Puccinia graminis* f. sp. *tritici*	AvrSr35	577	3	Yes	0	Non-effector	Non-effector

* TMD: Transmembrane Domain.

## Supplementary Materials

The following are available online at https://www.mdpi.com/2218-273X/10/5/712/s1. Table S1: Analysis of the PHI-Database. Table S2: Description of proteomes analyzed by EffHunter. Table S3: List of phytopathogens classified according to their lifestyle. Table S4: List of all fungi and oomycetes species classified according to their lifestyle. Supplementary data set S1: Positive data set of true effector proteins. Supplementary data set S2: Negative control set ABC transport proteins. Supplementary data set S3: Negative control set cytochrome P450 proteins. Supplementary data set S4: Negative control set major facilitator transporters (MFTS). Supplementary data set S5: Positive and negative controls pooled in a single database.
